# Mammalian TRP ion channels are insensitive to membrane stretch

**DOI:** 10.1242/jcs.238360

**Published:** 2019-12-10

**Authors:** Yury A. Nikolaev, Charles D. Cox, Pietro Ridone, Paul R. Rohde, Julio F. Cordero-Morales, Valeria Vásquez, Derek R. Laver, Boris Martinac

**Affiliations:** 1 Molecular Cardiology and Biophysics Division, Victor Chang Cardiac Research Institute, Sydney 2010, Australia; 2 Human Physiology, School of Biomedical Sciences and Pharmacy, University of Newcastle, Newcastle 2308, Australia; 3 Department of Physiology, College of Medicine, The University of Tennessee Health Science Center, Memphis 38163, USA; 4 St. Vincent's Clinical School, Faculty of Medicine, University of New South Wales, Sydney 2052, Australia

**Keywords:** TRP ion channels, TRPC6, Mechanosensitive channels, Ion channel reconstitution, Mechanotransduction

## Abstract

TRP channels of the transient receptor potential ion channel superfamily are involved in a wide variety of mechanosensory processes, including touch sensation, pain, blood pressure regulation, bone loading and detection of cerebrospinal fluid flow. However, in many instances it is unclear whether TRP channels are the primary transducers of mechanical force in these processes. In this study, we tested stretch activation of eleven TRP channels from six mammalian subfamilies. We found that these TRP channels were insensitive to short membrane stretches in cellular systems. Furthermore, we purified TRPC6 and demonstrated its insensitivity to stretch in liposomes, an artificial bilayer system free from cellular components. Additionally, we demonstrated that, when expressed in *C. elegans* neurons, mouse TRPC6 restores the mechanoresponse of a touch insensitive mutant but requires diacylglycerol for activation. These results strongly suggest that the mammalian members of the TRP ion channel family are insensitive to tension induced by cell membrane stretching and, thus, are more likely to be activated by cytoplasmic tethers or downstream components and to act as amplifiers of cellular mechanosensory signaling cascades.

## Abstract (precis)

**Summary:** Mammalian TRP ion channels are insensitive to membrane stretch, suggesting that they function as secondary – rather than primary – mechanotransducers in cellular mechanotransduction processes.

## INTRODUCTION

Transient receptor potential (TRP) ion channels constitute a superfamily of non-selective cationic channels permeable to Na^+^, Ca^2+^ and Mg^2+^, comprising 33 members found in mammals, of which 27 are found in humans (Clapham, 2003; Martinac and Cox, 2017). TRP channels are divided into six subfamilies: TRPC (canonical), TRPV (vanilloid), TRPM (melastatin), TRPP (polycystin), TRPML (mucolipin) and TRPA (ankyrin). These channels play a significant role in sensory physiology, with most of them contributing to cellular Ca^2+^ signaling and homoeostasis. All TRP channels are polymodally regulated and involved in various sensations in humans, including taste, temperature, pain, pressure and vision (Vriens et al., 2014; Julius, 2013). Multiple studies have demonstrated TRP channel involvement in mechanosensory transduction in mammals (Spassova et al., 2006; Wilson and Dryer, 2014; Spassova et al., 2004; Welsh et al., 2002; Quick et al., 2012), including most notably TRPA1 (Corey et al., 2004), TRPV4 (Loukin et al., 2010), TRPV2 (Muraki et al., 2003; Katanosaka et al., 2014), PKD2 (Narayanan et al., 2013), PKD2L1 (Sternberg et al., 2018), TRPC3 and TRPC6 (Nikolova-Krstevski et al., 2017; Quick et al., 2012; Seo et al., 2014).

Although TRP channels have been reported to be involved in mechanosensory processes, in most cases it remains controversial whether they are the primary transducers of mechanical force (Martinac and Cox, 2017; Christensen and Corey, 2007; Gottlieb et al., 2008). Mechanosensitivity of TRP channels is only understood well for no mechanoreceptor potential C (NOMPC; also known as TRPN) channels and the first such evidence came from a *Caenorhabditis elegans* study (Kang et al., 2010). NOMPC, whose homologs are not found in mammals, has also been shown to be activated by mechanical force pulling on the 29 ankyrin repeats that act as tethers associated with microtubules (Zhang et al., 2015). In *Drosophila*, NOMPC is involved in many mechanosensitive processes, including gentle touch in larvae (Yan et al., 2013). In contrast, other TRP channels, such as TRPV4, clearly respond to mechanical force (Teng et al., 2013; Servin-Vences et al., 2017) but their mechanisms for doing so are subject of debate (Teng et al., 2013; Loukin et al., 2010). Although the deletion of ankyrin repeats in TRPV4 does not eliminate the mechanosensitivity of the channel, it does reduce its sensitivity to hypotonicity. TRPV4 also contributes to mechanotransduction in chondrocytes and bone, and – in this context – the channel does not seem to respond to membrane stretch (Servin-Vences et al., 2017). Thus, although it is clear that TRP channels are components of mechanosensory systems, whether they are primary mechanosensors that respond to membrane stretch remains contentious.

Here, we systematically explore the stretch sensitivity for several representatives of all six mammalian TRP channel superfamily members that are heterologously expressed in HEK293T cells and most of which have been linked to mechanosensory transduction. In total, we investigate eleven TRP channels, including TRPC3, TRPC5, TRPC6, TRPM4, TRPM8, TRPV1, TRPV3, TRPV4, TRPA1, PKD2L1 and TRPML1. In addition, to examine the role the heterologous system itself has in channel activity, we tested TRPC6 sensitivity in response to membrane stretch in Chinese hamster ovary (CHO), mouse Neuro2a (N2a) and human HeLa cell lines, and purified channels reconstituted into liposomes and recording their activity by using the patch clamp technique. Our results indicate that none of the TRP channels we tested, including TRPV4, can be activated directly by membrane stretch.

To investigate whether mouse TRPC6 responds to mechanical stimuli *in vivo*, we examined the behavior of a transgenic *C. elegans*-expressed mouse TRPC6 on the background of a strain (i.e. *osm-9*) that is insensitive to mechanical stimuli. This *osm-9* strain lacks the gene encoding the *C. elegans* ortholog of the mammalian TRPV4 channel. We show that mouse TRPC6 is able to restore the nose-touch response in *osm-9* worms but requires diacylglycerol (DAG); suggesting that TRPC6 acts downstream of a mechanosensory pathway. We comprehensively illustrate the lack of stretch activation in the examined TRP channels and discuss our results in relation to the literature reporting TRP channels involvement in mechanosensory transduction processes.

## RESULTS

### TRP channels expressed in heterologous systems are insensitive to membrane stretch

**Fig. 1.**
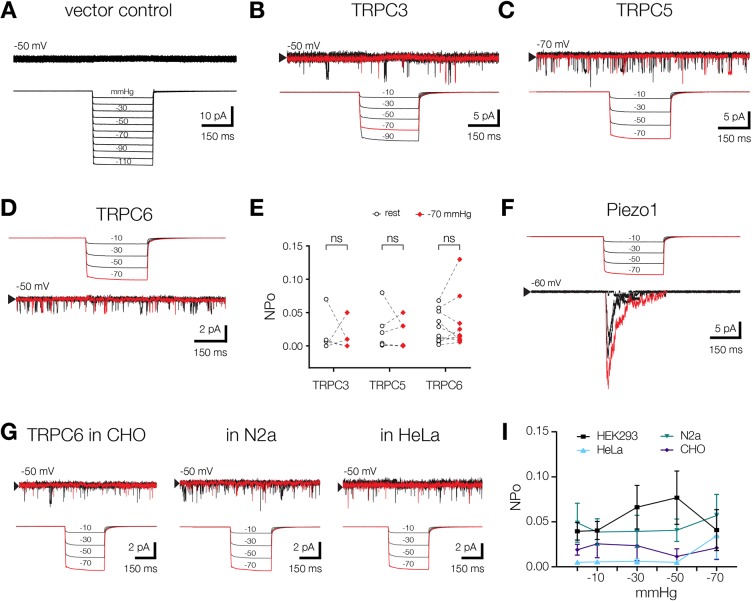
**TRPC ion channel subfamily members are insensitive to membrane stretch in mammalian cell lines.** (A) Application of negative pressure steps to HEK293T cells, transfected with the pIRES2-EGFP empty vector as a control. Mechanical stimulation was applied by high-speed pressure clamping in the recording electrode. A 300-ms pressure trace is shown under the current trace. (B) Application of negative steps of pressure leads to spontaneous channel activity of hTRPC3 overexpressed in HEK293T cells. Channel openings were recorded at the indicated voltage in cell-attached mode. The downward deflection of single channels represents outward current of the ions into the cell. The arrowhead indicates the baseline (closed state of the channel). (C) Application of pressure to mTRPC5 in cell-attached mode. (D) Application of pressure to hTRPC6 in cell-attached mode. (E) Quantification of the pressure effect on open probabilities (NPo) of TRPC channels expressed in HEK293T cells. For each channel NPo was calculated before and during pressure step. ns, not significant (paired *t*-test). (F) Example of the mechanosensitive ion channel hPiezo1 responding to the same pressure stimuli as those shown in C, D and G (i.e. they were in the range between −10 and −70 mmHg). hPiezo1 was expressed in HEK293T cells and recorded at indicated voltage in cell-attached mode. (G) Membrane stretch reaction of hTRPC6 overexpressed in CHO, N2a and HeLa cells. (I) Quantification of the NPo of hTRPC6 in the indicated mammalian cells in response to membrane stretch. Each value shows the mean±s.e.m.; *n*=8, 5, 4 and 3 (HEK293, CHO, N2a and HeLa cells, respectively). Note that the pipette voltage in A, B, D, F and G was −50 mV, whereas in C it was −70 mV. Negative pressure steps applied to the patch pipette are given in mmHg. The vertical scale bars in each figure indicate single-channel currents in pA (10^−12^ A). Note different scale values in each figure. The horizontal scale bars indicate recording time in ms.

Given the important role that TRP channels play in mechanosensory processes, we sought to determine whether these ion channels are sensitive to membrane stretch. We did this through the patch clamp technique, which is a electrophysiological technique is used to record activity of ion channels characterized by opening frequency of the single channels as well as ion currents flowing through the channels in response to a voltage and/or negative pressure (suction) applied to a patch clamp pipette. Initially, we recorded the activity of TRPC family members TRPC3, TRPC5 and TRPC6 in response to negative pressure pulses that had been applied to cell-attached patches for 300 ms (Fig. 1B–D). Channels were expressed in HEK293T cells and channel identity was confirmed by their ion conductance and, in the case of TRPC6, pharmacological properties (Figs S1 and S2, Table 1). We found that negative pipette pressure did not increase the probability of ‘open state’ (hereafter referred to as open probability) for any of the TRPC ion channels examined (Fig. 1E). As a positive control, we also expressed the well-known mechanosensitive channel Piezo1 (Coste et al., 2010) in HEK293T cells and found that stretch could elicit large inactivating currents that are characteristic of this ion channel (Fig. 1F). In addition, we also evaluated stretch activation of TRPC6 in three widely used heterologous expression systems, i.e. CHO, HeLa and N2a cells (Fig. 1G), to examine whether the cellular background plays a role in channel sensitivity in response to membrane stretch. In all three cell types, however, membrane stretch failed to increase the open probability of TRPC6 channel activity (Fig. 1I). Our data, thus, indicate that this member of the TRPC ion channel subfamily is insensitive to membrane stretch. Importantly, in these experiments we used wild-type HEK293T and N2a cells that express endogenous Piezo1 channels. Patches in which endogenous Piezo1 activity was detected were excluded from analysis.

**Table 1.**
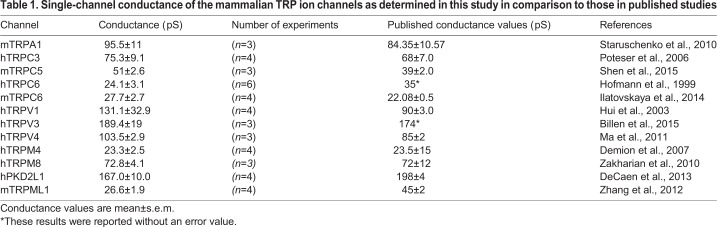
**Single-channel conductance of the mammalian TRP ion channels as determined in this study in comparison to those in published studies**

**Fig. 2.**
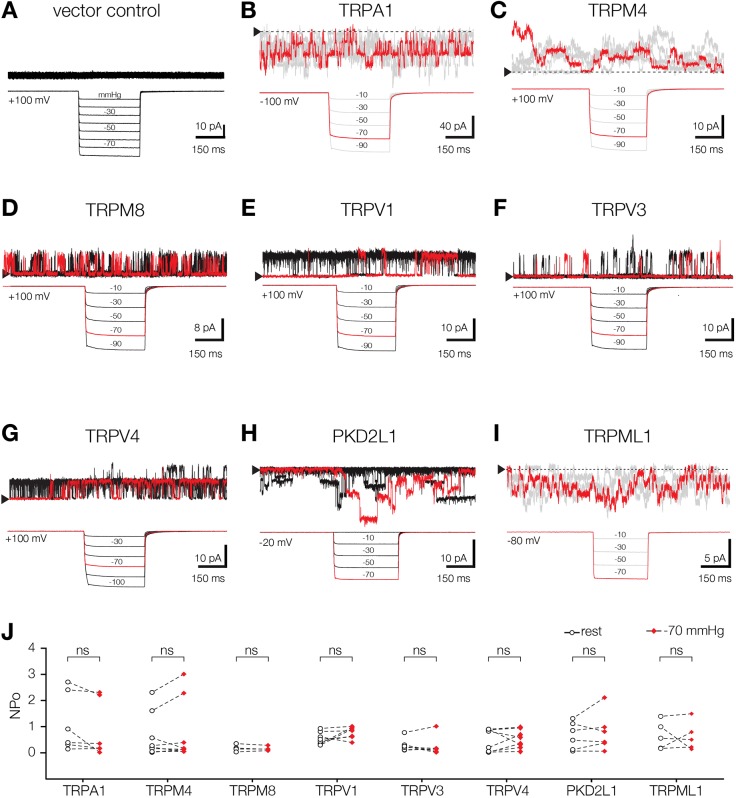
**Members of four subfamilies of TRP ion channels are insensitive to membrane stretch in HEK293T cells.** (A) Application of negative pressure steps to HEK293T cell, transfected with pIRES2-EGFP empty vector. (B) Stepwise application of negative pressure to spontaneously active mTRPA1 channels overexpressed in HEK293T cells. Multiple channel events were recorded in cell-attached mode. The downward deflection of single channels represents outward current. The baseline (channel is closed) with an arrow is shown. (C) Multiple single-channel events of hTRPM4 recorded during application of mechanical stimuli. The patch was excised into 2 mM Ca^2+^ bath solution at room temperature. Upward deflection represents outward current. (D) Application of pressure steps to hTRPM8 ion channel in cell-attached mode at room temperature. (E–G) Representative single-channel recording of TRPV family members. hTRPV1, hTRPV3 and hTRPV4 were recorded in cell-attach mode at room temperature. (H) Stretching TRPP channel family member hPKD2L1 in cell-attach mode in the HEK293T cells. (I) Multiple single-channel events of mTRPML1 (4A), surface expressing mutant, recorded during application of mechanical stimuli in HEK293T cells. The channel was recorded in inside-out patch clamp configuration. (J) Quantification of open probabilities (NPo) of TRP ion channel family members before (rest) and during stretch. ns, not significant (paired *t*-test). Other denotations are as in Fig. 1.

We applied a similar approach to test the stretch sensitivity of other TRP family members, i.e. TRPA1, TRPM4, TRPM8, TRPV1, TRPV3, TRPV4, PKD2L1 (TRPP2; formerly TRPP3) and TRPML1 (surface-expressing mutant) in HEK293T cells. We recorded the spontaneous resting activity of all tested channels that did not exhibit any change in response to membrane stretch (Fig. 2B–I). The channel identities were confirmed by their characteristic conductance (Figs S3, S4, S5 and Table 1). In each case, the channel open probability was not influenced by applied pipette pressure (Fig. 2J). Taken together, these data provide no evidence of stretch sensitivity in any of the TRP channels expressed in heterologous systems and examined in this study.

### TRPC6 channels reconstituted into liposomes are insensitive to bilayer stretch

**Fig. 3.**
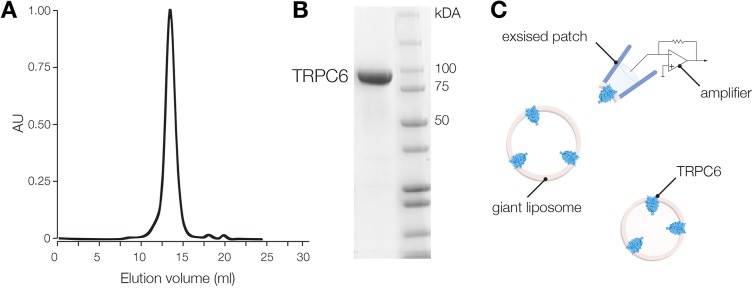
**Purification of mTRPC6 ion channels for liposome reconstitution.** (A) Size-exclusion chromatography profile of DDM-mTRPC6 protein complex corresponding to the tetramer state of the channel. AU, arbitrary unit. (B) SDS-PAGE showing stained mTRPC6 corresponding to the monomer state of the channel. The molecular mass standard is shown in kDa. (C) Illustration representing the patch excised from the liposome reconstituted with TRPC6.

A defining feature of inherently mechanically activated ion channels is that they can be activated by stretch in artificial lipid bilayers (Sukharev et al., 1994; Brohawn et al., 2014; Murthy et al., 2018; Martinac et al., 2010). Given the possibility that some components present in the heterologous systems inhibit stretch-activation of the TRP channel currents, we purified and reconstituted the TRPC6 channel protein into liposomes (Fig. 3) to examine the channel response to bilayer tension induced by applied pipette pressure. The purified TRPC6 protein appeared as a single monodisperse peak on a gel filtration column and as a single band on denaturing SDS-PAGE gels, indicating purity of the channel (Fig. 3A,B). For these experiments we used, in addition to WT, an N-terminal truncation (Δ94) of TRPC6, whose channel properties have been reported to be very similar to the WT channel (Azumaya et al., 2018). Previously, the Δ94 TRPC6 truncation was used for the structural determination of the TRPC6 cytoplasmic domain (Azumaya et al., 2018).

**Fig. 4.**
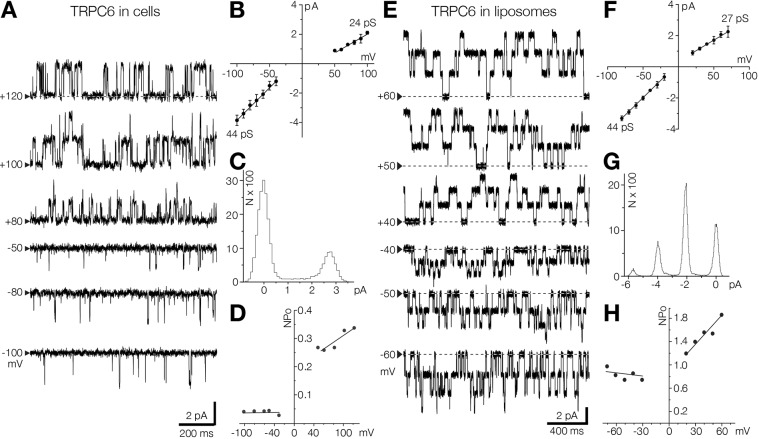
**Unidirectional incorporation of TRPC6 ion channel into the liposomal bilayer.** (A) Representative hTRPC6 single-channel recording at different voltages in inside-out patches from HEK293T cells. Black arrowheads indicate the position of the baseline current. Upward and downward single-channel deflections represent outward and inward current, respectively. (B) Current-voltage relationship for single TRPC6 channels in inside-out patch mode (*n*=6) from HEK293T cells. The single-channel conductance values are shown on the plot for inward (bottom left) and outward (top right) current: 44.6±4.7 pS and 24.1±3.1 pS respectively. (C) Amplitude histogram corresponding to the channel activity shown in A at +120 mV. (D) Open probability (NPo) of TRPC6 channel related to the patch voltages (mV) recorded in HEK293T cells. (E) Spontaneous activity of the Δ94 mTRPC6 channel in liposomes recorded at different voltages in the excised patch. Black arrowheads indicate the position of the baseline current. (F) Current voltage relationship of mTRPC6 recorded in liposomes with linear fitting (*n*=5). Single-channel conductance values for inward and outward are 44.5±0.6 pS (bottom left) and 27.7±2.7 pS (top right), respectively. (G) Amplitude histogram corresponding to the channel activity shown in E, at −50 mV. (H) The relationship between voltage (mV) and open probability (NPo) of the TRPC6 recorded in liposomes. Other denotations are as in Fig. 1.

In addition to WT TRPC6 channels, we also observed spontaneous openings of Δ94 TRPC6 channels expressed in liposomes, with conductance values similar to those of TRPC6 channel expressed in cells (Fig. 4). Remarkably, the WT TRPC6 channel was significantly more active in liposomes than in cells (Fig. 4B,C,F,G). TRPC6 has been reported to be an outward rectifier – i.e. passing current more easily out of the cell – in whole cell mode (Tang et al., 2018 preprint). When recording single-channel activities in cell-attached mode, we found that positive holding potentials slightly decreased single-channel conductance (Fig. 4B) and increased the open probability of the channel (Fig. 4D), which explains the outward-rectifying currents observed in the whole-cell recordings. We also found that, like in cells, open probability and conductance of the recorded channels in liposomes were modulated by voltage (Fig. 4 B–D,F–H). Conductivity of full-length TRPC6 reconstituted into liposomes was comparable to that of its truncated version used in this study (Fig. S6). Collectively, our data demonstrate that TRPC6 assembled into a pore-forming oligomer in liposomes, where it exhibits biophysical properties comparable to those exhibited by channels expressed in cells. The observation that TRPC6 is inwardly rectifying in liposomes, strongly suggests that the channel incorporates unidirectionally into the artificial bilayer – with the cytoplasmic domain facing the external bath solution (Fig. 3C), similar to what has been reported for several other reconstituted ion channels (Ajouz et al., 2000; Kloda et al., 2007; Nomura et al., 2015).

**Fig. 5.**
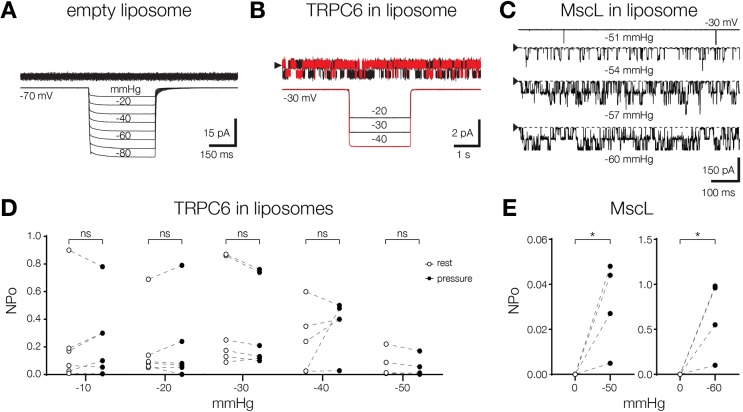
**TRPC6 reconstituted in liposomes is insensitive to bilayer stretch.** (A) Application of stretch to the liposome reconstituted with no protein control. (B) Representative trace of a constitutively active mTRPC6 channel that has been subjected to the negative pressure steps in the liposomes. The channel was recorded at indicated voltage in excised patch. The arrowhead indicates the closed state of the channel. (C) Application of the stretch to the MscL ion channel reconstituted into the liposomes, similar to the experiment shown in B. The downward deflections represent the inward current. The pressure values are shown under each trace. Black arrowheads indicate the position of the baseline current. (D) Summary of NPo change upon application of the stretch to the TRPC6 channel reconstituted in the liposomes (*n*=6). For each applied pressure the NPo was calculated before and during pressure step. (E) Change of NPo upon application of negative pressure to MscL, similar to the experiment shown in D (*n*=4). *P<0.05; ns, not significant, paired *t*-test. Other denotations are as in Fig. 1.

We further examined the mechanosensitivity of TRPC6 reconstituted into liposomes by applying mechanical stretch to excised patches. We found that spontaneously active TRPC6 was insensitive to the application of negative pressure (Fig. 5B) and its open probability was unaffected by stretching the liposome bilayer (Fig. 5D). In contrast, stretching the well-characterized mechanosensitive channel MscL (Sukharev et al., 1994; Häse et al., 1995) reconstituted into liposomes, activated the channel and increased its open probability (Fig. 5C,E). Application of stretch to empty liposome patches in control experiments did not elicit any currents (Fig. 5A). These results demonstrate that, activation of TRPC6 is unaffected by the changes in bilayer tension, comparable to the results obtained with the channels expressed in heterologous cell systems.

### TRPC6 restores nose-touch response in *osm-9* worms but requires diacylglycerol

**Fig. 6.**
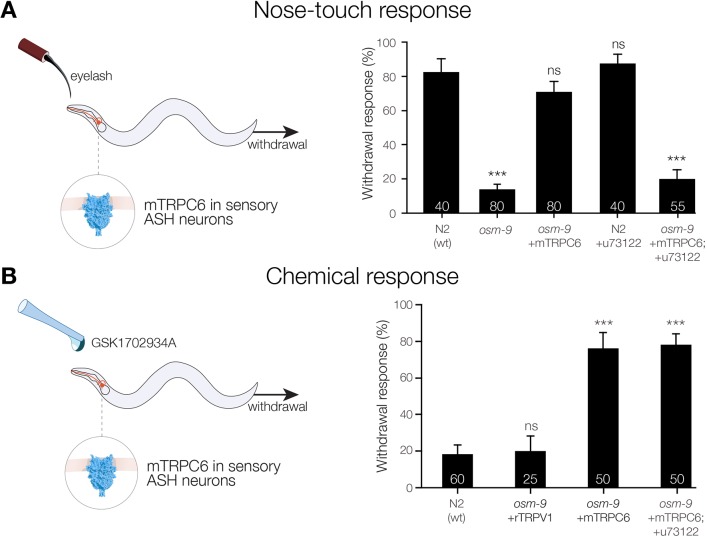
**TRPC6 restores touch responses in *osm-9* worms but requires diacylglycerol.** (A) Schematic representation of the nose-touch response assay in worms. A *C*. *elegans* *osm-9* mutant expressing *Mus musculus* (m)TRPC6 in sensory ASH neurons is subjected to eyelash stroke. (B) Schematic representation of chemical response test in a *C*. *elegans* *osm-9* mutant expressing mTRPC6 in sensory ASH neurons. The freely moving worm is subjected to a drop of the mTRPC6 activator GSK1702934A (50 μM). The respective bar graphs of the right show the quantification (mean±s.e.m.) of the withdrawal responses upon eyelash stroke or application of GSK1702934A. *n* values for each condition are indicated within the bars. ***P<0.001; ns, not significant, Mann–Whitney test.

TRPC6 has been reported numerous times to be involved in mechanosensory processes (Nikolova-Krstevski et al., 2017; Quick et al., 2012; Seo et al., 2014; Yamaguchi et al., 2017). However, whether TPRC6 is a primary mechanotransducer still remains to be determined. We implemented *C. elegans* as an *in vivo* model to determine the role of TRPC6 in mechanotransduction. In this animal model, it is well-established that mammalian TRPV4 can restore touch sensitivity in *osm-9* worms (a mutant strain insensitive to nose-touch stimuli; Fig. 6A) when expressed in ASH sensory neurons, i.e. the neurons that are required in *C. elegans* for a wide range of avoidance behaviors – and by stroking the nose of the worm with an eyelash (Caires et al., 2017; Liedtke et al., 2003). OSM-9 is the *C*. *elegans* orthologue of mammalian TRPV4; noteworthy, OSM-9 acts downstream of the DEG-1 channel in the ASH-mediated mechanical response (Colbert et al., 1997; Geffeney et al., 2011).We expressed *Mus musculus* (m)TRPC6 (no TRPC6 ortholog is encoded in the *C. elegans* genome) in ASH sensory neurons to determine whether TRPC6 can recover the touch response of *osm-9* worms (Fig. 6A). We found that, upon mechanical stimulation, mTRPC6 could restore the withdrawal response of *osm-9* worms, similar to that in the wild-type (N2) worms (Fig. 6A), establishing that the channel is functional in ASH neurons (Fig. 6A). However, when reducing the DAG content by feeding phospholipase C (PLC) inhibitor (u73122) to the worm (Fig. S7A), mTRPC6 lacks the ability to recover touch sensitivity. It is noteworthy that feeding worms an inactive PLC inhibitor analog (u73343) did not affect TRPC6-mediated response (Fig. S7C). PLC enzymes are known to obtain DAG from the inner membrane monolayer, which in turn activates TRPC6 via GPCR pathway (Hofmann et al., 1999). Therefore, our results suggest that mTRPC6 is acting downstream of a mechanosensory pathway that generates DAG but not as a direct mechanosensor. To test whether the DAG content also modulates the chemically induced response of TRPC6, we challenged mTRPC6 transgenic worms with the TRPC6 specific agonist GSK1702934A (Fig. 6B) (Xu et al., 2013). We found that GSK1702934A elicited dose-dependent robust withdrawal responses (Fig. S7B) in mTRPC6-expressing but not in wild-type or TRPV1 transgenic worms (Fig. 6B). Unlike the mechanical response, inhibition of PLC did not affect the withdrawal behavior mediated by chemical activation of mTRPC6 (Fig. 6B), since the agonist directly activates the channel.

## DISCUSSION

In this study we investigated the mechanosensitivity of eleven TRP channels from all six families of the TRP ion channel superfamily heterologously expressed in HEK293T cells, examining their activity in response to stretching of the cell membrane in patch clamp experiments. In addition to TRPC3, TRPC5 and TRPC6, we also examined TRPM4, TRPM8, TRPV1, TRPV3, TRPV4, TRPA1, PKD2L1 and TRPML1. We focused further on TRPC6 by examining the stretch sensitivity of this channel when expressed in three other cell lines, i.e. CHO, HeLa and N2a cells. We also purified the TRPC6 channel protein and reconstituted it into liposomes to examine its stretch sensitivity in isolation. The key observation from these experiments is that none of the examined TRP channels exhibited increased activation in response to a membrane stretch of 300 ms, indicating that none of these channels are directly activated by membrane tension. As a control, we used the well-characterized mechanosensitive Piezo1 and MscL channels, which under the same experimental conditions exhibited robust stretch-activated ion currents in cell and liposome membrane patches, respectively.

Overall, our results contrast with the results reported in many studies describing TRP channel activation by membrane stretching, which has been reported numerous times to activate TRP channels: TRPV4 (Loukin et al., 2010), TRPA1 (Kwan et al., 2006), PKD1/PKD2 (TRPP2) (Nauli et al., 2003), TRPC1 (Maroto et al., 2005), TRPM4 (Morita et al., 2007), TRPC5 (Shen et al., 2015) and TRPC6 (Spassova et al., 2006; Wilson and Dryer, 2014; Anderson et al., 2013; Yamaguchi et al., 2017; Dyachenko et al., 2009). Notably, inherent mechanosensitivity – which would suggest TRP channels to be primary mechanosensors – was never provided for any of these channels. In agreement with our findings, some studies do not support the inherent mechanosensitivity of TRPC6 (Gottlieb et al., 2008), TRPV4 (Servin-Vences et al., 2017) and TRPM4 (Constantine et al., 2016). Given that cells are highly heterogeneous systems, with diverse protein and lipid composition, the cellular environment can be expected to play an essential role in the function of mechanosensitive, i.e. stretch-activated, ion channels. Therefore, considering the heterogeneity of expression systems as a possible cause for the discrepancies between different reports, we tested the mechanosensitivity of TRPC6 channels in several other types of cell as well as in proteoliposome patches. Despite the absence of the cellular environment in proteoliposome patches we were unable to record TRPC6 activation in response to membrane stretch. This result is consistent with our results concerning the other TRP channels whose membrane-stretch sensitivity we investigated in HEK293T cells. Consequently, given our results and the evidence provided by many studies reporting the involvement of different types of TRP ion channels in cellular mechanotransduction processes (Christensen and Corey, 2007; Lin and Corey, 2005; Patel et al., 2010; Yin and Kuebler, 2010; Eijkelkamp et al., 2013), we conclude that most mammalian TRP channels are not stretch-sensitive primary mechanosensors but are more likely to be secondary membrane receptors involved in downstream mechanosensory signaling cascades. Indeed, we showed that mouse TRPC6 can act downstream of a mechanosensory pathway in *C. elegans*; however, it requires DAG to recover the mechanical response.

We cannot, however, completely exclude the possibility that some of these TRP channels, nevertheless, function as primary mechanoreceptors if – like NOMPC channels – they are tethered directly to the cytoskeleton or extracellular matrix. As secondary downstream mechanoreceptors, these channels can contribute to either an increase of intracellular Ca^2+^ (e.g. TRPC3, TRPC6, TRPV1, TRPV4, PKD2L1) or the depolarization of the membrane potential through inward Na^+^ and/or Ca^2+^ currents (e.g. TRPM4, TRPA1). In some cases, where TRP channels are Ca^2+^-dependent, they may be directly downstream of Ca^2+^ permeable mechanosensitive channels such as Piezo1. In other instances, transduction of the mechanical force to the channel may occur through other mechanosensing proteins. For example, GPCRs can be activated by mechanical force (Xu et al., 2018) and produce a host of secondary messengers (e.g. cAMP, cGMP, Ca^2+^, DAG) that activate TRP channels through ligand–channel interactions. One scenario for Gq-coupled receptors is the cleavage of PIP_2_ to produce DAG through activation of PLC (Kadamur and Ross, 2013). In this case, there are three possible scenarios for how this may stimulate TRP channel opening. First, DAG might act as a ligand opening the channel. Second, PIP_2_ might have an inhibitory influence, thus its removal will allow the channel to open (Qin, 2007). Third, the shape change instigated by conversion of PIP_2_ to DAG might generate local membrane curvature in the vicinity of a TRP channel causing it to open, as demonstrated for TRP and TRPL channels in *Drosophila melanogaster* (Hardie and Franze, 2012). Such a scenario has been proposed for TRPC6 channels (Spassova et al., 2006), given that local membrane curvature can generate enough stress in the membrane bilayer to activate mechanosensitive channels (Bavi et al., 2016). Indeed, as has been previously shown (Winn et al., 2005), we were also able to activate TRPC6 channels in response to the DAG analogue 1-oleoyl-2-acetyl-sn-glycerol (OAG) (Fig. S2). This indicates that ion channel mechanosensitivity exists in different forms, with local membrane curvature being one of them. Although membrane stretch and local membrane curvature are mechanical stimuli that activate mechanosensitive channels, such as MscL, MscS, TREK-1, TRAAK and Piezo1 (Bavi et al., 2017), this does not seem to be the case for other ion channels, such as TRPC6, which may be able to differentiate between these two kinds of mechanical stimulus. Further to this point, it is important to note that even homologues of the prototypical mechanosensitive channel MscL exhibit a different sensitivity to membrane stretch and curvature, despite their high degree of sequence similarity (Mukherjee et al., 2014). Thus, it is eminently feasible that channel structure influences stretch and curvature sensitivity of an ion channel (Cox et al., 2019).

Further to our results that demonstrate insensitivity to membrane stretch of mammalian TRP channels, we also demonstrated, for the first time, a successful functional reconstitution of TRPC6 ion channels in a model lipid system without any other protein components present. TRPC6 ion channels were spontaneously active and exhibited high open probability in liposomes. They were ∼40 times more active in liposomes compared to within HEK293T cell patches. Spontaneous ion channel activities upon reconstitution into artificial lipid bilayers have also previously been reported for Piezo1 channels (Syeda et al., 2016; Coste et al., 2012). Furthermore, the lack of TRPC6 responsiveness in liposomal membranes further confirms that not all proteins reconstituted in lipid bilayers are gated by membrane stretch.

In conclusion, we have demonstrated that mammalian members of different subfamilies of TRP channels are insensitive to membrane stretch. This does not preclude their involvement in cellular mechanotransduction processes but does suggest, in many cases, that they do not represent the primary mechanotransducers. If any one of them is, in fact, a primary mechanotransducer, then its mechanosensory abilities are likely to be dependent on auxiliary transmembrane proteins or tethers that link them to structural scaffold proteins. However, we showed that activation of transgenically expressed mouse TRPC6 in *C. elegans* is DAG dependent and were able to activate TRPC6 *in vitro* with OAG. Both reagents have been proposed to generate local bilayer curvature in the vicinity of the channel. From these results we conclude that the mechanosensitivity of TRP channels can be different compared to that of other well-known mechanosensitive ion channels, including MscL and Piezo1. This suggests that controversies regarding the mechanosensitivity of the TRP ion channels originate from the fact that different mechanical stimuli, such as membrane stretch, shear force or local membrane curvature, do not affect all mechanosensitive channels in the same way. The structure of some channels, including the TRP ion channels, might have, in fact, evolved to discriminate between different mechanical stimuli based on their interactions with other membrane and cellular components.

## MATERIALS AND METHODS

### Cell culture and transient transfection

HEK293T, N2a, HeLa and CHO cells were used for heterologous expression of TRP channels. Cells were grown in Dulbecco's modified Eagle medium (DMEM) with 10% FBS, then transferred on 2 ml Petri dishes for transfection. Lipofectamine 3000 Transfection Reagent (Thermo Fisher Scientific) kit was used according to manufacturer instructions: 1–3 μg of plasmid (Table S1), 5 μl of Lipofectamine 3000, and 5 μl of P3000 were mixed in 250 μl Opi-MEM (Gibco) for 5 min and then added to cells for 24 h incubation. Some plasmids were mixed with 0.5 μg GFP (pIRES2-EGFP) to enable selection of transfected cells. The surface-expressing TRPML1 mutant TRPML1-L15L/AA-L577L/AA (4A) was used for patch clamp recordings. All plasmids were verified by Sanger Sequencing.

### Expression and purification of TRPC6

Protocol for mTRPC6 purification was used from a previous publication (Azumaya et al., 2018). Two versions of mTRPC6 were purified: 94 aa truncated (Δ94 mTRPC6) and the full-length (WT) protein. Both versions were 8His-maltose binding protein (MBP) tagged on the N- terminus. DNA constructs were cloned into the pFastbac1 plasmid. Recombinant baculovirus was generated (Bac-to-Bac expression system; Invitrogen) and transfected into Sf9 cell culture (800 ml) for 72 h. Cell pellets were resuspended and disrupted with a homogenizer (Avestin) in a solution containing 36.5 mM sucrose, 50 mM Tris, 4 mM TCEP (pH 8), and protease inhibitors: 1 mM PMSF, 1 mg/ml pepstatin, 3 mg/ml aprotinin, and 3 mg/ml leupeptin. Unbroken cells were removed by centrifugation at 8000 ***g*** for 15 min. Supernatants were further ultracentrifuged (Beckman) at 100,000 ***g*** for 30 min at 4°C. Pellets were solubilized in 26 mM DDM (Anatrace), 150 mM NaCl, 10% Glycerol, 50 mM HEPES, 2 mM TCEP (pH 7.4), and protease inhibitors by rotating for 2 h. Then the mixtures were ultracentrifuged at 150,000 ***g*** for 45 min, and the supernatants were incubated with amylose resin (New England Biolabs) for 3 h. The resins were loaded onto the column and washed with 10 volumes of 0.5 mM DDM, 150 mM NaCl, 10% glycerol, 50 mM HEPES, and 2 mM TCEP (pH 7.4). The proteins were eluted with same solution supplemented with 20 mM maltose. The MBP tags were cleaved with ProTEV Plus protease (Promega) at 4°C overnight. Proteins were further purified by Superose 6 10/300 GL column (GE Healthcare) running in a solution containing 150 mM NaCl, 20 mM HEPES, and 2 mM TCEP (pH 7.4). The peak fractions corresponding to the tetrameric mTRPC6 channel were collected for proteoliposome reconstitution.

### Proteoliposome reconstitution

Liposome reconstitution of the TRPC6 and MscL proteins was carried out using modified method as previously published (Nomura et al., 2015). L-α-Phosphatidylcholine from soybean (Sigma-Aldrich, P5638) was stored in chloroform that was evaporated using nitrogen before use. The lipids were resuspended in 200 mM KCl and 5 mM HEPES (pH 7.2) at a final concentration of 10 mg/ml. The mixture was vortexed for 5 min and sonicated for 15–30 min until the liposome suspension became translucent. Purified mTRPC6 was mixed with the lipids (1:100 protein-lipid w:w) and incubated for 1 h at room temperature. Bio-Beads SM2 resin (Bio-Rad, 1523920) was used to remove detergent by incubating (15 mg/ml) with the protein lipid mixture for 3 h at room temperature. The supernatant was ultracentrifuged at 150,000 ***g*** (Beckman) for 30 min. The pellet was resuspended in 30 µl of solution containing 200 mM KCl and 5 mM HEPES (pH 7.2), then spotted onto the clean glass slide, and dried in the desiccator overnight at room temperature. The next day, samples were hydrated with the same solution and further incubated overnight at 4°C. Proteoliposomes (5 µl) were added to the recording chamber and incubated for 15 min before patching. For TRPC6: bath solution contained: 140 mM NaCl, 10 mM KCl, 0.2 mM CaCl_2_, and 5 mM HEPES (pH 7.4). Patch pipettes were filled with the same solution. After GΩ seal formation the patch was always excised. Wild-type *E. coli* MscL ion channel was purified, as reported previously (Häse et al., 1995), using immobilized metal affinity chromatography and reconstituted into the liposomes similarly to TRPC6. MscL was recorded in symmetrical bath and pipette solution containing 200 mM KCl, 40 mM MgCl_2_, and 5 mM HEPES (pH 7.2).

### Electrophysiology

Channel activity was directly recorded in cells in Petri dishes within 48 h after transfection. Glass recording pipettes (Drummond, 100 μl), were pulled with a vertical puller (Narishige PP-83). A cell-attached or inside-out patch mode was used to record single-channel activity. Resistance of the patch pipettes was in the range of 2–5 MΩ. GΩ seals with the cell membrane were aided by applying ∼10 mmHg negative pressure. Seal formation was monitored by pipette current. A high-speed pressure clamp (ALA Scientific Instruments), connected to the patch pipette allowed rapid pressure application. Single-channel events were recorded with a patch-clamp amplifier Axopatch 200B (Molecular Devices) at 10 kHz sampling rate and filtered at 1–2 kHz with a Digidata^®^ 1440 Digitizer (Molecular Devices) using pCLAMP v10.3 software (Molecular Devices). The voltage values shown in the figures are holding potentials. All patch-clamp recordings were conducted at room temperature (∼22°C). Cells were recorded in the bath solution: 145 mM NaCl, 5 mM KCl, 1 mM CaCl_2_, and 3 mM HEPES (pH 7.2). The pipette solution contained 90 mM CsCl, 50 mM CsF, 5 mM HEPES, and 4 mM EGTA (pH 7.2 adjusted with NaOH). For TRPML1 recordings bath solution also contained 1 mM MgCl_2_; pipette solution contained 140 mM NaCl, 5 mM KCl, 1 mM MgCl_2_, 1 mM EGTA, and 10 mM HEPES (pH 7.4). 1-Oleoyl-2-acetyl-sn-glycerol (OAG) (Avanti) and SKF96365 (Cayman Chemical) were dissolved in DMSO and were added to the external solution with final concentration 30 μM and 10 μM, respectively.

### *C. elegans* strains and behavioral assays

Worm strains were cultured as previously described (Brenner, 1974). The wild-type (N2) *C. elegans* strain was obtained from the Caenorhabditis Genetics Center, which is funded by the NIH Office of Research Infrastructure Programs (P40 OD010440). Transgenic worms were obtained using the MosSCI method (Frøkjær-Jensen et al., 2012). The GN132 strain was a gift from Dr Miriam B. Goodman (Stanford University). A complete list of strains is presented in Table S2. The chemical response test was performed at 21°C by placing a drop containing the TRPC6 agonist GSK1702934A (50 μM; Tocris Bioscience) or control buffer (M13 buffer: 30 mM Tris–HCl pH 7.0, 100 mM NaCl, 10 mM KCl, with 1% ethanol) in front of moving young adult hermaphrodites as described previously (Hart, 2006; Vásquez, 2020). The nose-touch response test was performed by stroking the worm with an eyelash as previously described (Geffeney et al., 2011). Drop or touch trials that elicited reversals of motion were scored as withdrawal responses. The PLC inhibitor u73122 or its inactive analog u73343 (Tocris Bioscience) dissolved in DMSO (10 mM) were added to the nematode growth medium (NGM) to reach a 10 µM concentration.

### Liquid chromatography–mass spectrometry

Control- and u73122-treated worms (500 worms per condition) were rinsed three times with M9 buffer containing 22 mM KH_2_PO_4_, 42 mM Na_2_HPO_4_, 86 mM NaCl, and 1 mM MgSO_4_. Worms were frozen in liquid nitrogen to further extract diacylglycerols (DAGs) at the Lipidomics Core Facility at Wayne State University.

### Data analysis

Data were analysed using the Clampfit 10.3 software (Molecular Devices) and GraphPad Prism 7. Mean single-channel amplitudes for each voltage were acquired by fitting Gaussian curves to the current-amplitude histograms. The conductance values for single channels were obtained by calculating the slope of the current voltage plots. The relative open probability (NPo) was obtained using the single-channel search function in Clampfit. For stretch experiments NPo values were calculated before and upon mechanical stretch. For NPo/voltage plots, open probabilities were calculated for 5 s periods at each voltage. Statistical analyses were carried out using paired *t*-tests when comparing two groups. Data were reported as mean±s.e.m.;*P<0.05 was considered statistically significant. Statistical analyses of Worm withdrawal were carried out using the Mann–Whitney test.

## Supplementary Material

Supplementary information

Reviewer comments
