# Supplementary material for: Mammalian TRP ion channels are insensitive to membrane stretch
Source: J Cell Sci. 2019 Dec 10;132(23):jcs238360. doi: 10.1242/jcs.238360 (PMC6918743; doi:10.1242/jcs.238360)
Supplement: Supplementary information [file joces-132-238360-s1.pdf]

Table s1. Key resources

Recombinant DNA	*Source*
Human Piezo1- pIRES2-EGFP	Philip Gottlieb, SUNY Buffalo
Mouse TRPA1- pcDNA5/FRT/TO	Alexander Staruschenko, Medical College of Wisconsin,
Human TRPC3- pcDNA3.1	Philip Gottlieb, SUNY Buffalo
Mouse TRPC5- pcDNA6	Xiaoqiang Yao, The Chinese University of Hong Kong
Human TRPC6- pIRES2-EGFP	Eric Honoré, IPMC
Human TRPV1- pEYFP-N3	Linlin Ma, The University of Queensland
Human TRPV3- pcDNA5/FRT/TO	Sara Baratchi, RMIT University
Human TRPV4- pcDNA 5/FRT/TO	Peter McIntyre, RMIT University
Human TRPM4- pCAGGS/IRES GFP	Veit Flockerzi, Saarland University
Human TRPM8- pEF5/FRT/V5	Sara Baratchi, RMIT University
Human PKD2L1- pIRES2-EGFP	Paul DeCaen, Northwestern University
Mouse TRPML1(4A)- pEGFP/C2	Youxing Jiang, University of Texas

Table s2. List of *C. elegans* strains

Strain	*Genotype*
Wild type	N2
GN132	*osm-10(rtIs27) III; osm-9(ky10) IV; rtIs27 (Posm-10::GFP X)*
COP1493	*knuSi749 [pnu1336 (osm10p::rtrpv1-wt-optimized::tbb-2u, unc-119(+))] II; unc-119(ed3) III; osm-9(ky10) IV; rtIs27 (Posm-10::GFP X)*
COP1830	*knuSi791 [pnu1668 (osm10p::mTRPC6-wt-optimized::tbb-2u, unc-119(+))] II; unc-119(ed3) III; osm-9(ky10) IV; rtIs27 (Posm-10::GFP X)*

Journal of Cell Science • Supplementary information

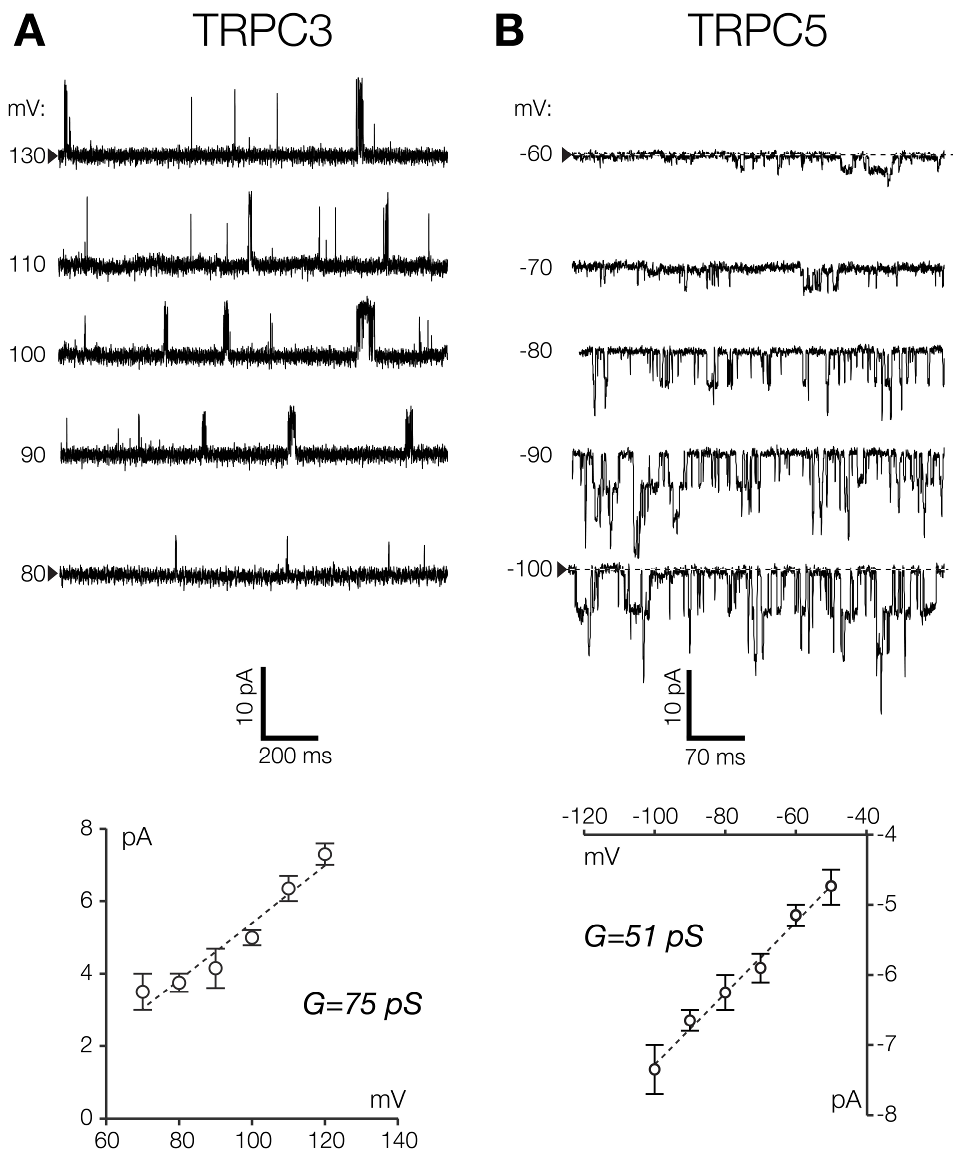

Figure s1. Single channel properties of TRPC3 and TRPC5 ion channels in HEK293T cells

(A) (B) Representative hTRPC3 and mTRPC5 single channel recording at different voltages in cell-attached patch from HEK293T cells. Black arrows indicate the position of the baseline current. Downward single channel deflections represent inward current. At the bottom: current-voltage relationship for single channels of TRPC3 (N=4) and TRPC5 (N=3), with indicated single channel conductance (mean ± s.e.m.): 75.3±9.1 pS and 51±2.6 pS.

Journal of Cell Science • Supplementary information

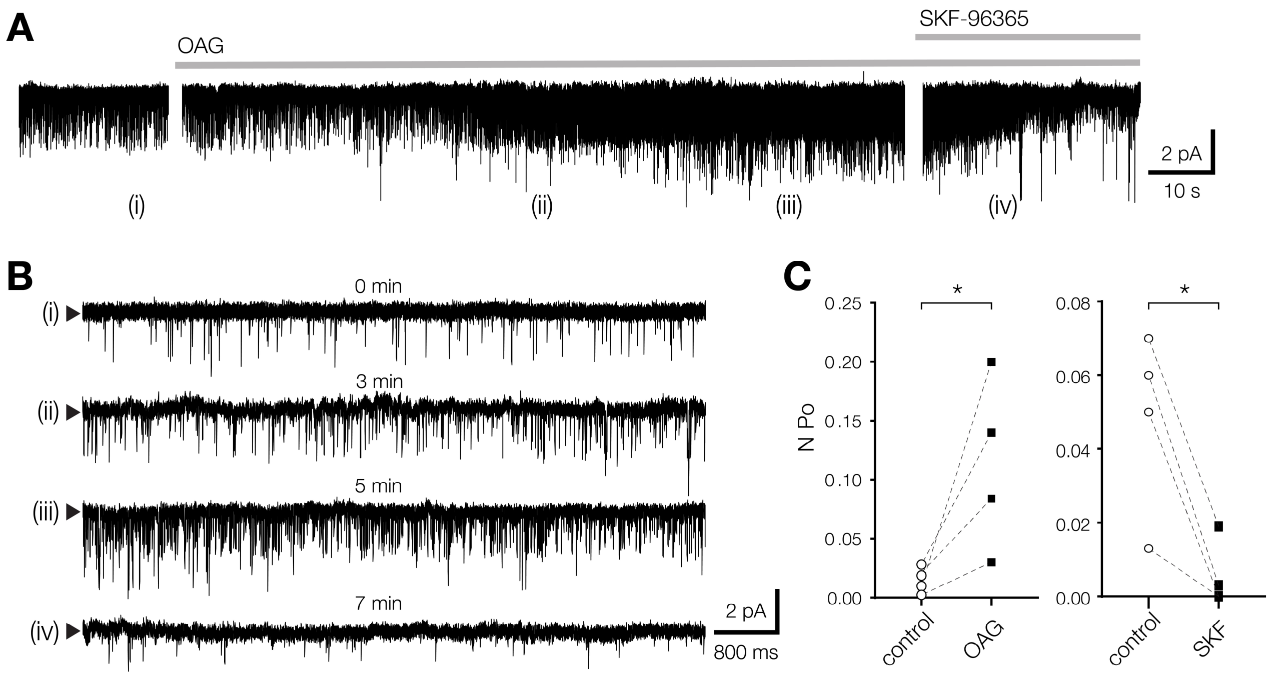

Figure s2. Pharmacology of TRPC6 ion channels

(A) Application of agonist OAG (30 µM) and antagonist SKF-96365 (10 µM) to TRPC6 single channel activity in inside-out mode at -50 mV. (B) Enlarged segments of TRPC6 channel activity from (A) (C) Graph summarizing the channel open probability (NPo) before and after application of OAG and SKF-96365. *P < 0.05, paired t-test

Journal of Cell Science • Supplementary information

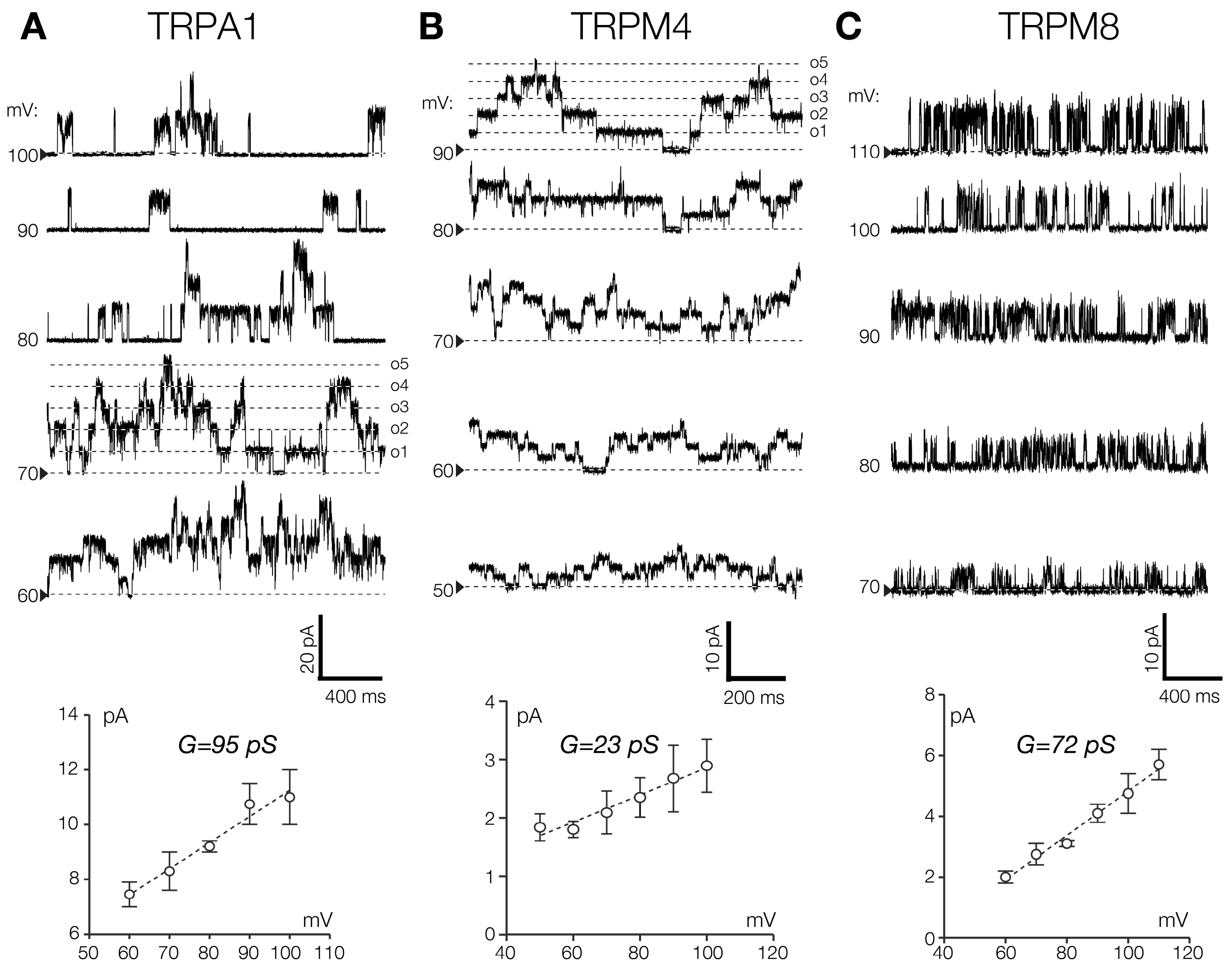

Figure s3. Single channel properties of TRPA1, TRPM4, and TRPM8 ion channels in HEK293T cells

(A) (B) (C) Representative mTRPA1, hTRPM4, and hTRPM8 single channel recording at different voltages in cell-attached patch recorded at room temperature. Black arrows indicate the closed state of the channels. Upward single channel deflections represent outward current. At the bottom: single conductance values (mean±s.e.m.) with current-voltage relationships for single channels of TRPA1 (N=3), TRPM4 (N=4) and TRPM8 (N=3): 95.5±11 pS, 23.3±2.5 pS, and 72.8±4.1 pS respectively.

Journal of Cell Science • Supplementary information

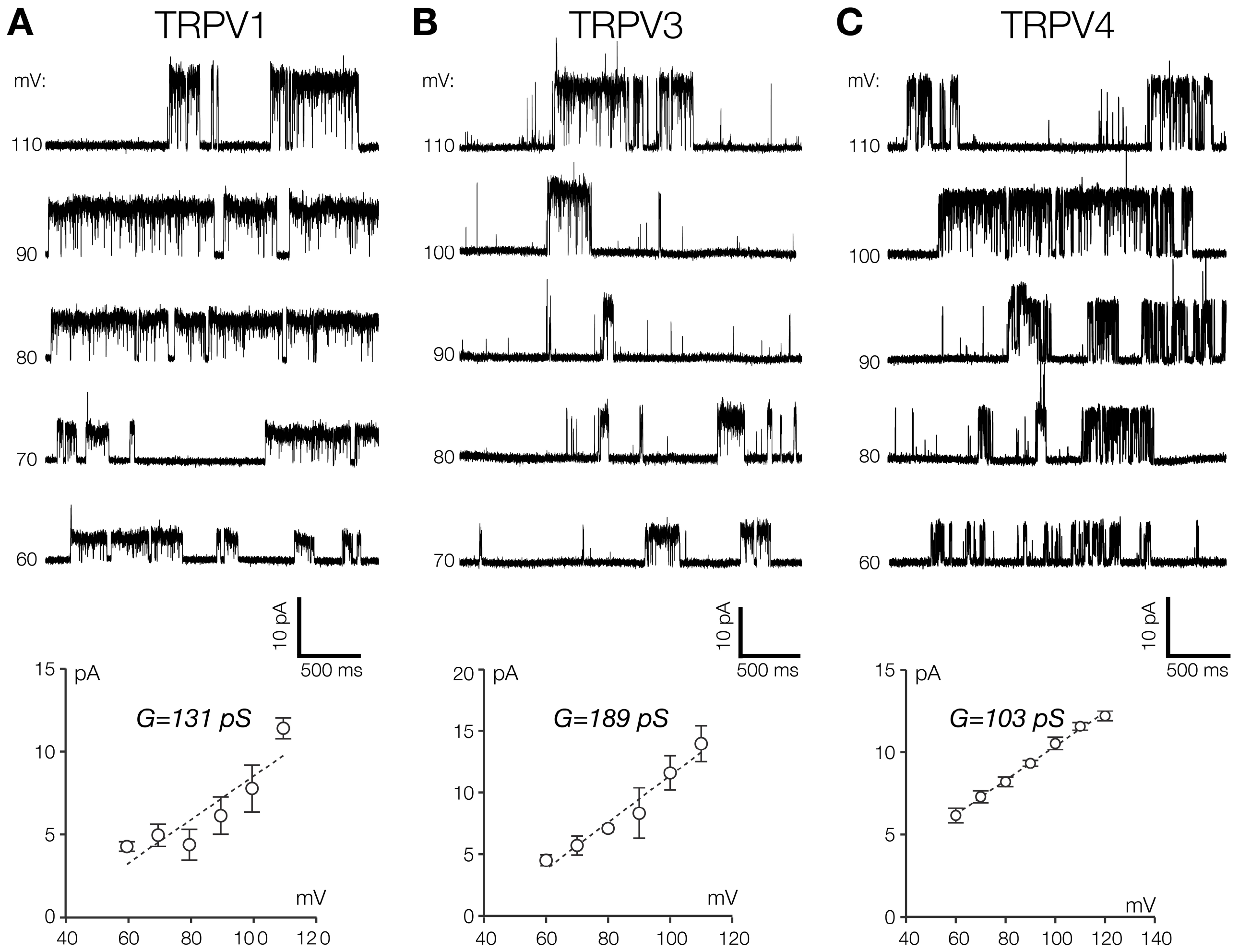

Figure s4. Single channel properties of TRPV subfamily channel members in HEK293T cells

(A) (B) (C) Single channel recordings of hTRPV1, hTRPV3, and hTRPV4 at different voltages in cell-attach patch recorded at room temperature. Upward single channel deflections represent outward current. At the bottom: fittings with single conductance values (mean±s.e.m.) of TRPV1 (N=4), TRPV3 (N=3), and TRPV4 (N=3): 131.1±32.9 pS, 189.4±19 pS, and 103.5±2.9 pS respectively.

Journal of Cell Science • Supplementary information

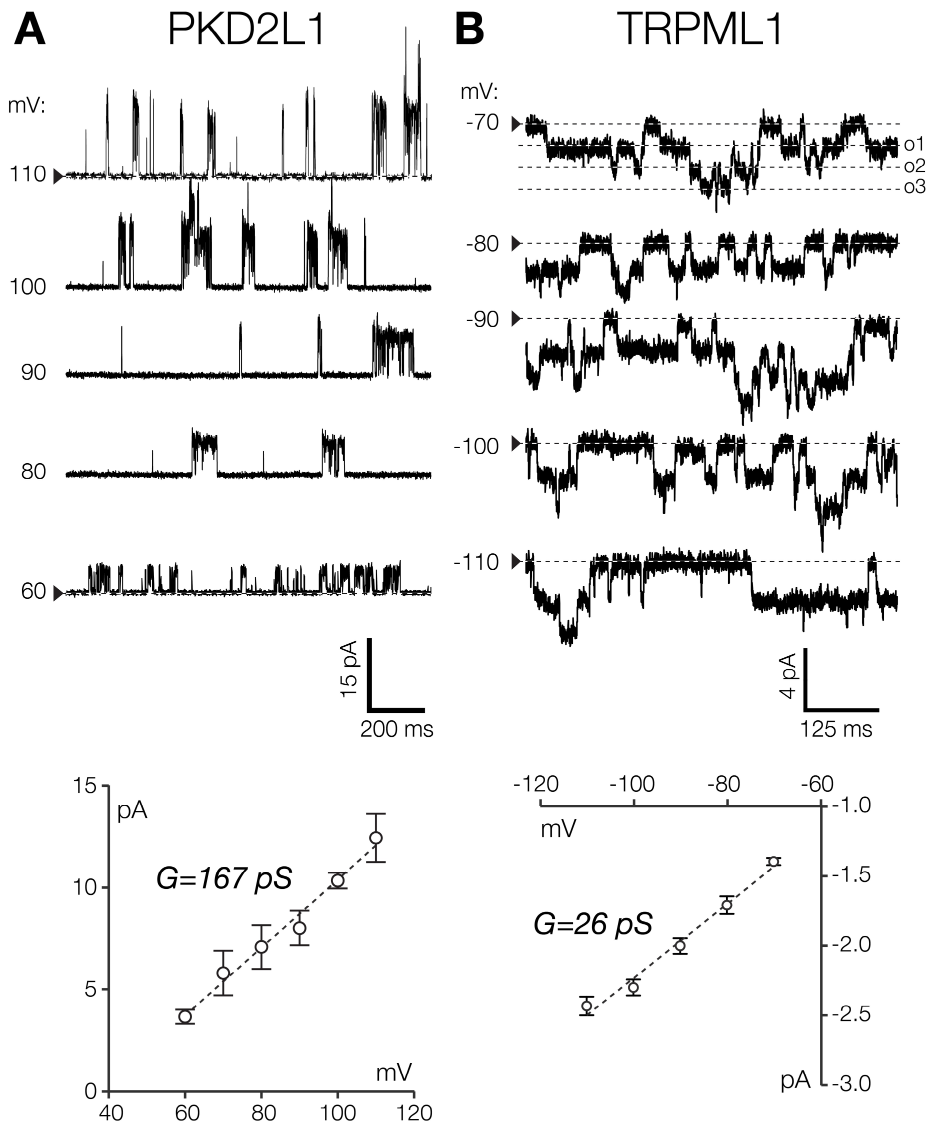

Figure s5. Single channel properties of PKD2L1 and TRPML1 channels in HEK293T cells

(A) (B) Single channel recordings of hPKD2L1 and mTRPML1 (4A), surface expressing mutant, at different voltages. PKD2L1 was recorded in cell-attached configuration, TRMPL1 was recorded in inside-out configuration. At the bottom: fittings with single conductance values (mean±s.e.m.) of PKD2L1 (N=4) and TRPML1 (N=4): 167.0±10 and 45±2 pS.

Journal of Cell Science • Supplementary information

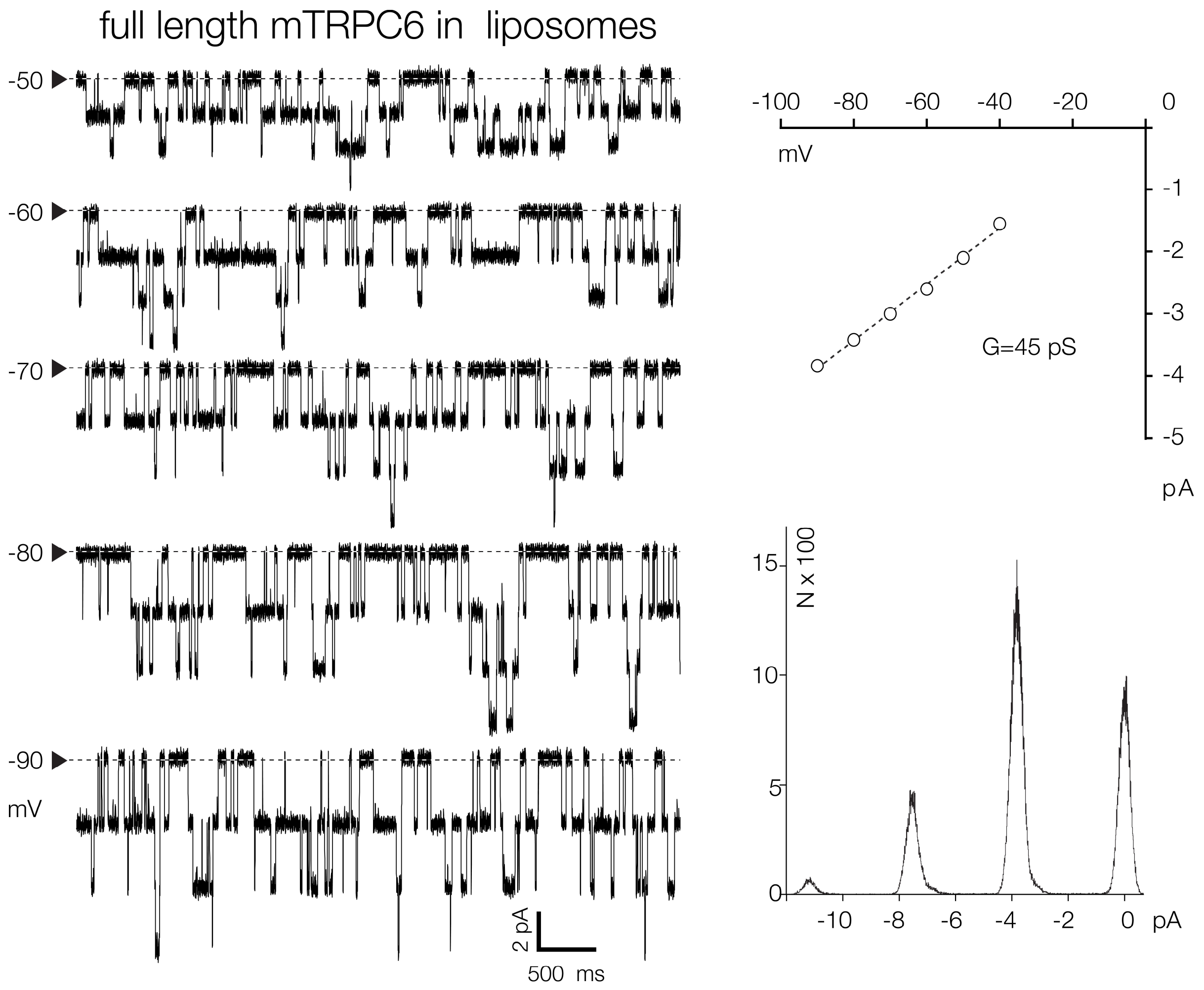

Figure s6. Single channel properties of full length TRPC6 recorded in liposomes

Spontaneous activity of the full length mTRPC6 channel in the liposomes recorded at a range of voltages in excised patch. On the right: current-voltage relationship with the linear fitting and amplitude histogram corresponding to the channel activity at -90 mV. N=1.

Journal of Cell Science • Supplementary information

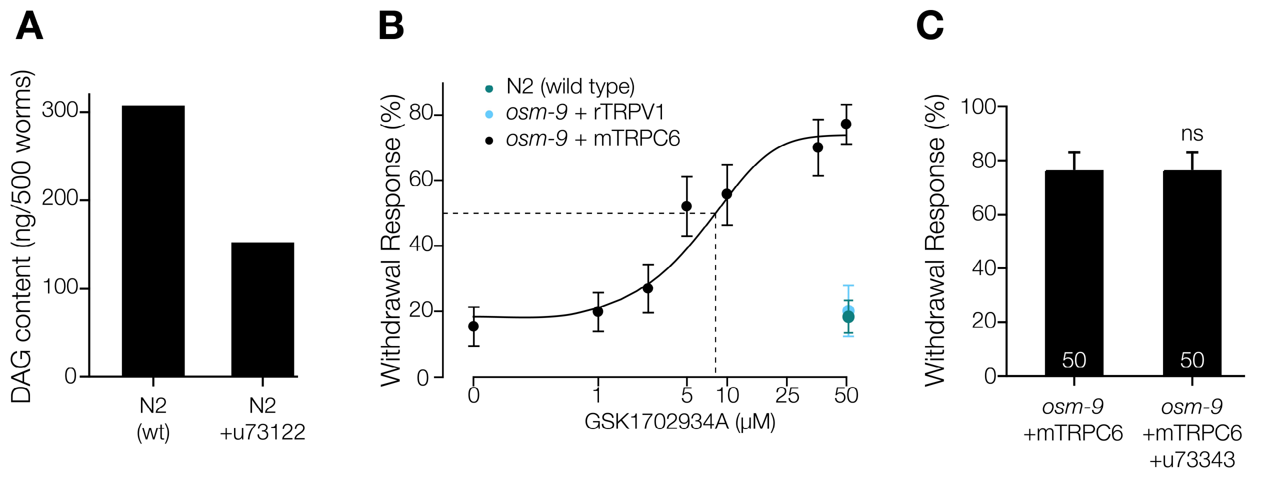

Figure s7. Related to Figure 6

(A) Diacylglycerol (DAG) content in control *vs.* PLC inhibitor (u73122) treated worms as determined by LC-MS. (B) Dose-response profile of TRPC6 agonist GSK1702934A for wild type (N2), TRPC6, and TRPV1 expressing *osm-9* worms. For each value (mean ± s.e.m.) n ≥ 25. EC_{50} = 7.01 ± 9.7 μM. (C) Worms (*osm-9* strain) expressing mTRPC6 were fed with an inactive PLC inhibitor analog (u73343) and then subjected to touch response test.

Journal of Cell Science • Supplementary information
