# Supplementary material for: Mammalian TRP ion channels are insensitive to membrane stretch
Source: J Cell Sci. 2019 Dec 10;132(23):jcs238360. doi: 10.1242/jcs.238360 (PMC6918743; doi:10.1242/jcs.238360)
Supplement: Reviewer comments [file joces-132-238360_review_history.pdf]

Mammalian TRP ion channels are insensitive to membrane stretch

Yury Nikolaev, Charles D. Cox, Pietro Ridone, Paul R. Rohde, Valeria Vasquez, Julio Cordero Morales, Derek R. Laver and Boris Martinac
DOI: 10.1242/jcs.238360

Editor: Guangshuo Ou

Review timeline
Original submission: 21 August 2019
Editorial decision: 13 September 2019
First revision received: 22 October 2019
Accepted: 29 October 2019

Original submission

First decision letter

MS ID#: JOCES/2019/238360

MS TITLE: TRP ion channels are insensitive to membrane stretch

AUTHORS: Yury Nikolaev, Charles D. Cox, Pietro Ridone, Paul R. Rohde, Valeria Vasquez, Julio Cordero Morales, Derek R. Laver and Boris Martinac
ARTICLE TYPE: Research Article

We have now reached a decision on the above manuscript.

To see the reviewers' reports and a copy of this decision letter, please go to: https://submit-jcs.biologists.org and click on the 'Manuscripts with Decisions' queue in the Author Area. (Corresponding author only has access to reviews.)

As you will see, the reviewers raise a number of substantial criticisms that prevent me from accepting the paper at this stage. They suggest, however, that a revised version might prove acceptable, if you can address their concerns. If you think that you can deal satisfactorily with the criticisms on revision, I would be pleased to see a revised manuscript. We would then return it to the reviewers.

Please ensure that you clearly highlight all changes made in the revised manuscript. Please avoid using 'Tracked changes' in Word files as these are lost in PDF conversion.

I should be grateful if you would also provide a point-by-point response detailing how you have dealt with the points raised by the reviewers in the 'Response to Reviewers' box. Please attend to all of the reviewers' comments. If you do not agree with any of their criticisms or suggestions please explain clearly why this is so.

Reviewer 1

Advance summary and potential significance to field

In this paper, the authors examined 10 TRP channels for their sensitivity to mechanical stretch in HEK293T cells, but found that none of them is sensitive to stretch. They have also tested one TRP

© 2019. Published by The Company of Biologists under the terms of the Creative Commons Attribution License (https://creativecommons.org/licenses/by/4.0/).

channel TRPC6 in an in vitro reconstituted system, and again obtained a negative result. Finally, using C. elegans ASH neurons as a vehicle, they showed that TRPC6 acts downstream of an endogenous mechanosensor and PLC to mediate nose touch behavioral response.
Over the years, many TRP channels have been reported to be mechanosensitive but none (with one exception: TRPN1) has been shown to be mechanically gated.
Though this has been pointed out in literature, some groups in the field are still arguing that those TRP channels are mechanically gated. This paper will help to further clarify this point, and should be of value to the field.
Technically, this work is also nicely done. I am happy to support its publication after the authors address the following comments:

Comments for the author

Specific points:

1) TRPN1 is the only TRP channel that has thus far been demonstrated as a mechanically-gated channel. The first such evidence in fact came from a C. elegans study (TRP-4/TRPN1) by Kang et al 2010 Neuron. The cited Drosophila work was reported afterwards. Please fix the citation.

2) Title: because one TRP channel is known to be stretch-sensitive, the title needs to be revised to reflect this. In addition, the authors tested 10 TRP channels but not all the 28 TRPs. The current title is too strong.

3) U73122 feeding exp is interesting. A negative control is needed: U73243 (inactive analog of the former), to make sure that it is not like that worms just became unhealthy after drug treatment.

4) What is osm-9? This needs to be explained in the paper (it is a TRPV channel).

5) More experimental details need to be included for the C. elegans work for example, how the behavior was tested and scored, how the data was quantified, what buffer was used, temperature, etc?

Reviewer 2

Advance summary and potential significance to field

Previous studies have suggested that some TRP channels might serve as mechanotransduction channels involved in many physiological processes such as touch, blood pressure regulation. However, in the literature there are lack of report showing that TRP channels themselves are intrinsically mechanosensitive. In this study, Martinac and colleagues have used multiple approaches to systematically examine the stretch-sensitivity of 10 different TRP channels and found none of them directly respond to membrane stretch when heterologously expressed. Furthermore, they have purified and reconstituted TRPC6 into liposome, which were subjected to patch clamp studies. In contrast to the mechanosensitive MscL channel, the reconstituted TRPC6 did not respond to mechanical stretch of the membrane. They additionally showed that TRPC6 is indirectly involved in the mechanosensory response in the worm. Together, their studies suggest that TRP channels are insensitive to membrane stretch, which might help to clarify the role of TRP channels in previously suggested mechanobiology.

Comments for the author

The authors may consider the following suggestions to improve the manuscript.
1. Given that NOMPC, the mechanosensitive TRP channel in fly, has been clearly shown to respond to membrane stretch by Yuh-Nung Jan's group, the current title of the manuscript appears to be

© 2019. Published by The Company of Biologists under the terms of the Creative Commons Attribution License (https://creativecommons.org/licenses/by/4.0/).

misleading. It might be changed to "Mammalian TRP ion channels are insensitive to membrane stretch".

2. For Fig. 1 and Fig. 2, a vector-transfected negative control should be included. If it is possible, it will also be important to include NOMPC as a positive control.

First revision

Author response to reviewers' comments

Reviewer 1 Advance summary and potential significance to field

In this paper, the authors examined 10 TRP channels for their sensitivity to mechanical stretch in HEK293T cells but found that none of them is sensitive to stretch. They have also tested one TRP channel TRPC6 in an in vitro reconstituted system, and again obtained a negative result. Finally, using C. elegans ASH neurons as a vehicle, they showed that TRPC6 acts downstream of an endogenous mechanosensor and PLC to mediate nose touch behavioral response. Over the years, many TRP channels have been reported to be mechanosensitive, but none (with one exception: TRPN1) has been shown to be mechanically gated. Though this has been pointed out in literature, some groups in the field are still arguing that those TRP channels are mechanically gated. This paper will help to further clarify this point and should be of value to the field. Technically, this work is also nicely done. I am happy to support its publication after the authors address the following comments:

Response: We are grateful for the overall positive comments of the Reviewer on our manuscript.

Reviewer 1 Comments for the author

Specific points:

1)TRPN1 is the only TRP channel that has thus far been demonstrated as a mechanically gated channel. The first such evidence in fact came from a C. elegans study (TRP-4/TRPN1) by Kang et al 2010 Neuron. The cited Drosophila work was reported afterwards. Please fix the citation.

Response: We cite the paper by Kang et al. on page 10 in our revised manuscript.

2)Title: because one TRP channel is known to be stretch-sensitive, the title needs to be revised to reflect this. In addition, the authors tested 10 TRP channels but not all the 28 TRPs. The current title is too strong.

Response: We agree with the Reviewer's comment and have changed the title to "Mammalian TRP ion channels are insensitive to membrane stretch" as suggested by the second Reviewer. We also examined stretch activation of the TRPML1 channel, a member of another subfamily of the mammalian TRP channels, which thus justifies the title of our manuscript. The results of the TRPML1 experiments are shown in Fig. 2I and Fig. S5.

3) U73122 feeding exp is interesting. A negative control is needed: U73243 (inactive analog of the former), to make sure that it is not like that worms just became unhealthy after drug treatment.

Response: Figure 6B shows that U73122 feeding does not affect worm's behaviour, as osm- 9 +mTRPC6 worms fed with U73122 have a ≈80% response when challenged by TRPC6 specific agonist GSK1702934A. Nevertheless, we have included in the revised manuscript the negative control suggested by the reviewer. As shown in Fig. S7C, we found that U73343 does not decrease osm-9+mTRPC6 worms mechanical response.

© 2019. Published by The Company of Biologists under the terms of the Creative Commons Attribution License (https://creativecommons.org/licenses/by/4.0/).

4)What is osm-9? This needs to be explained in the paper (it is a TRPV channel).

Response: OSM-9 is the C. elegans ortholog of the mammalian TRPV4 ion channel, which has now been explained on p. 4 and p. 9 of the revised manuscript.

5)More experimental details need to be included for the C. elegans work, for example, how the behavior was tested and scored, how the data was quantified, what buffer was used, temperature, etc?

Response: We apologize for this oversight and now have included more details in the Methods' section on p. 14 of the revised manuscript.

Reviewer 2 Advance summary and potential significance to field

Previous studies have suggested that some TRP channels might serve as mechanotransduction channels involved in many physiological processes such as touch, blood pressure regulation. However, in the literature there are lack of report showing that TRP channels themselves are intrinsically mechanosensitive. In this study, Martinac and colleagues have used multiple approaches to systematically examine the stretch-sensitivity of 10 different TRP channels and found none of them directly respond to membrane stretch when heterologously expressed. Furthermore, they have purified and reconstituted TRPC6 into liposome, which were subjected to patch clamp studies. In contrast to the mechanosensitive MscL channel, the reconstituted TRPC6 did not respond to mechanical stretch of the membrane. They additionally showed that TRPC6 is indirectly involved in the mechanosensory response in the worm. Together, their studies suggest that TRP channels are insensitive to membrane stretch, which might help to clarify the role of TRP channels in previously suggested mechanobiology.

Response: We gratefully acknowledge also this Reviewer's positive comments on our manuscript.

Reviewer 2 Comments for the author

The authors may consider the following suggestions to improve the manuscript.
1. Given that NOMPC, the mechanosensitive TRP channel in fly, has been clearly shown to respond to membrane stretch by Yuh-Nung Jan's group, the current title of the manuscript appears to be misleading. It might be changed to "Mammalian TRP ion channels are insensitive to membrane stretch".

Response: We thank the Reviewer for this helpful suggestion. We have changed the title accordingly.

2. For Fig. 1 and Fig. 2, a vector-transfected negative control should be included. If it is possible, it will also be important to include NOMPC as a positive control.

Response: The results of a vector-transfected negative control in Fig. 1A and Fig. 2A have been included in both figures and described on p. 23 of the revised manuscript as requested by the reviewer. Concerning inclusion of NOMPC results, Yuh-Nung Jan's group has convincingly shown that NOMPC is activated by mechanical force pulling on microtubules via the ankyrin tether (Zhang et al., 2015). Moreover, the same study also showed that equipping a non-mechanosensitive K+ channel with the NOMPC ankyrin repeats converted it into an MS channel. Given the evidence, we believe that inclusion of the Piezo1 and MscL channel recordings in cells and liposomes in our study provides an adequate and sufficient positive control for our TRP channel results.

© 2019. Published by The Company of Biologists under the terms of the Creative Commons Attribution License (https://creativecommons.org/licenses/by/4.0/).

Second decision letter

MS ID#: JOCES/2019/238360

MS TITLE: Mammalian TRP ion channels are insensitive to membrane stretch

AUTHORS: Yury Nikolaev, Charles D Cox, Pietro Ridone, Paul R Rohde, Valeria Vasquez, Julio Cordero Morales, Derek R Laver, and Boris Martinac
ARTICLE TYPE: Research Article

I am happy to tell you that your manuscript has been accepted for publication in Journal of Cell Science, pending standard ethics checks.

Reviewer 1

Advance summary and potential significance to field

The authors have addressed all my comments. Happy to support its publication.

Comments for the author

Congratulations!

Reviewer 2

Advance summary and potential significance to field

The authors have systematically examined the mechanosensitivity of various mammalian TRP channels and found that they are not direct mechanosensitive. This study might help to clarify the confusion in the field regarding whether mammalian TRP channels might function as mechanotransduction channels.

Comments for the author

In the revised manuscript, the authors have properly addressed my comments. I would like to recommend the publication of the paper.

© 2019. Published by The Company of Biologists under the terms of the Creative Commons Attribution License (https://creativecommons.org/licenses/by/4.0/).
